# Tumor–Microenvironment Interaction: Analysis of Mast Cell Populations in Normal Tissue and Proliferative Disorders of the Canine Prostate

**DOI:** 10.3390/vetsci6010016

**Published:** 2019-02-13

**Authors:** Sabrina Vanessa Patrizia Defourny, Mariarita Romanucci, Valeria Grieco, Gina Rosaria Quaglione, Chiara Santolini, Leonardo Della Salda

**Affiliations:** 1 Faculty of Veterinary Medicine, University of Teramo, 64100 Teramo, Italy; sdefourny@unite.it (S.V.P.D.); mromanucci@unite.it (M.R.); 2 Department of Veterinary Medicine, University of Milan, 20154 Milan, Italy; valeria.grieco@unimi.it; 3 Unità Ospedaliera Complessa, Anatomia patologica, Ospedale G. Mazzini, 64100 Teramo, Italy; gina.quaglione@aslteramo.it; 4 Veterinary Practitioner, 63812 Montegranaro, Italy; chiasan@alice.it

**Keywords:** tumor microenvironment, mast cells, prostate cancer, dog

## Abstract

Mast cells (MCs) are involved in angiogenesis, tissue remodeling and immunomodulation in several human and animal tumors, although their exact role is still controversial. Since no information is available in canine prostate carcinoma (PC) and normal prostate tissues, the aims of this study were to evaluate the possible correlations between MC distribution, molecular expression and microvessel density (MVD) in normal prostatic tissue and proliferative disorders of the canine prostate. All samples (6 normal, 15 benign prostate hyperplasia-BPH, 8 PC) were stained with Toluidine Blue and immunohistochemically evaluated for tryptase, c-Kit (CD117) and CD31. Mast cell density (MCD) and MVD were quantified by the hot-spot method. MCD was significantly increased in periglandular/peritumoral areas, when compared with intraglandular/intratumoral areas, in all groups (*p* = 0.03). C-Kit expression was strongly associated with PC (ρ = 0.75 *p* = 0.03), whereas positive correlation between tryptase and c-Kit expression (ρ = 0.64 *p* = 0.01) was observed in periglandular areas of BPH. MVD showed a correlation with MCD in BPH (ρ = 0.54 *p* = 0.04). Our data support the importance of c-Kit in regulating MC proliferation. The predominant location of MCs in peritumoral areas of canine PC was similar to the human counterpart, in which PC cells are supposed to produce substances attracting MCs to the tumor microenvironment.

## 1. Introduction

Recent studies in human and veterinary medicine have focused on the interactions between tumor cells and the surrounding microenvironment, in order to better describe the characteristics of cancer [1,2,3,4,5,6]. Tumor microenvironment is an evolving concept that defines the behavior of cancer not only by the study of tumor cells alone, but also in association with the surrounding background that tumor cells need for survival, growth, proliferation and metastatic spread [7]. Tumor microenvironment is an interconnected and dynamic network that includes: cancer cells, stromal tissue (immune cells, fibroblasts, blood vessels, neural cells), secreted soluble and insoluble factors such as chemokines, cytokines and extracellular matrix [4,6,8,9,10]. 

As in many other tumors, a tumor-stroma interaction has been observed in prostate cancer. The consequence of such interaction is a desmoplastic response or reactive stroma, with aberrant growth and morphologic changes of the stroma and connective tissues surrounding neoplastic cells [11]. Indeed, several cells of the innate and adaptive immune system, such as macrophages, mast cells (MCs), lymphocytes, neutrophils, and natural killer cells, are stromal components that can promote prostate cancer development [11,12,13,14].

MCs are involved in angiogenesis, tissue remodeling and immunomodulation in several human tumors, by synthesizing and releasing potent mediators, cytokines, proteases and growth factors, such as vascular endothelial growth factor (VEGF), Matrix Metalloproteinases (MMP), nerve growth factor (NGF), fibroblast growth factor (FGF)-2, stem cell factor (SCF), histamine, heparin, and tryptase [12,15,16,17,18,19]. MCs can also remodel the tumor microenvironment through degranulation and release of these cytokines and proteases [20]. There are many studies concerning the role of MCs in human prostate cancer, although the exact role of MCs is still controversial [21,22,23,24,25]. In fact, MCs can exert pro- or anti-tumor effects depending on the tumor type and microenvironment [21,22,23,24,25]. However, no information is available for canine prostate cancer. 

The dog is one of few domestic species to develop spontaneous prostate cancer and its role as a possible animal model has been suggested [26,27]. Even if the incidence of the disease is considerably higher in men than in dogs, most prostate tumors in both species are carcinomas [26,28]. In addition, similar to humans, affected dogs often develop pulmonary, regional lymph node, and/or osteoblastic bone metastases [26,28,29,30]. The androgen-independence of canine prostate cancer also offers the unique opportunity to use the canine model for studying advanced, hormone-refractory prostate cancer in men [27,29].

An increasing tendency exists towards the use of cancer therapies targeting not only tumor cells, but also the tumor microenvironment [6], with particular interest focused on MCs [31,32,33]. Thus, the aim of this study was to evaluate MC populations and their possible relationship with microvessel density (MVD) in normal, hyperplastic and neoplastic canine prostatic tissues in order to identify possible new strategies for prostate cancer therapy in both canine and human patients.

## 2. Materials and Methods

### 2.1. Case Selection

Twenty-nine formalin-fixed paraffin-embebbed (FFPE) canine prostate tissues were selected from the archives of the Veterinary Pathology Unit of the Faculty of Veterinary Medicine, University of Teramo (6 normal, 15 benign prostate hyperplasia—BPH, 2 prostate carcinoma—PC) and of the Faculty of Veterinary Medicine, University of Milan (6 PC). 

Normal tissues were collected during necropsy from animals that spontaneously died from causes not related to prostatic diseases, whereas BPH and PC samples were collected during necropsy (for all BPH and 5 PC cases) or by means of surgical excisional biopsy (for 3 PC cases). The median age of dogs was 0.34, 7.50, and 9.00 years for normal, BPH and PC group, respectively. All samples were obtained from sexually intact dogs. 

### 2.2. Histology and Immunohistochemistry

All samples were stained with hematoxylin-eosin (H/E) and Toluidine Blue (TB) (0.1% TB solution in 30% ethanol) for the evaluation of MC presence and distribution. PC cases were classified according to Palmieri et al. [34].

Immunohistochemistry was performed using primary antibodies (Abs) specific for tryptase (1:300, Code M7052, Monoclonal Mouse Anti-Human, DAKO, Glostrup, Denmak), c-Kit (1:500 CD-117, Code A4502, Polyclonal Rabbit Anti-Human, DAKO, Glostrup, Denmark) and CD31 (1:25, Monoclonal Mouse Anti-Human, Code M0823, DAKO, Glostrup, Denmak). All Abs were previously validated for canine tissues [35,36,37].

Deparaffinized and rehydrated sections were incubated with 3% H_2_O_2_ in deionized water for 30 min at room temperature to inhibit endogenous peroxidase activity and then rinsed in 0.05 M Tris-buffered saline (TBS) pH 7.6 for 5 min. Antigen retrieval was performed by heat treatment in pH 6.0 citrate buffer, for tryptase and c-Kit, and TRS Hi 50x (DAKO) buffer for CD31, in a water bath for 2 h at 80 °C. To reduce non-specific binding, slides were then incubated with Bovine Serum Albumin (BSA) 5% in PBS for 20 min and subsequently with normal goat serum (NGS) (code: S-1000, Vector laboratories, Inc., Burlingame, CA, USA) for 20 min at room temperature before overnight incubation with the primary Ab. Immune complexes were treated with secondary biotinylated anti-mouse (1:200 code: BA-9002, Vector laboratories, Inc., Burlingame, CA, USA) or anti-rabbit (1:200 code: BA-1000 Vector laboratories, Inc., Burlingame, CA, USA) Abs at room temperature for 30 min and subsequently detected using Vectastain elite ABC Horseradish Peroxidase (HRP) kit (code: PK-6100 Vector laboratories, Inc., Burlingame, CA, USA) for 30 min at room temperature. Peroxidase activity was detected by a 5min application of 3-3′-diaminobenzidine 0.8% solution (code: D5905, Sigma–Aldrich, St. Louis, Mo, USA, USA) with 0.1% H_2_O_2_ followed by counterstaining with Mayer’s hematoxylin, before dehydrating and mounting.

### 2.3. Double Immunofluorescence

Double immunofluorescence was carried out to investigate the relationship between MCs and blood vessels. Tissue samples were treated as described for the immunohistochemical procedure. Primary Abs specific for tryptase (1:300, Code M7052, Monoclonal Mouse Anti-Human, DAKO, Glostrup, Denmak) and Von Willebrand Factor (1:1000 Polyclonal Rabbit Anti-Human, Code A0082, DAKO, Glostrup, Denmark) were applied overnight at 4 °C. The first secondary, biotinylated anti-mouse Ab (for Tryptase; 1:200 code: BA-9002, Vector laboratories, Inc., Burlingame, CA, USA) was applied and incubated for 30 min at room temperature, and slides were then incubated with Texas Red-conjugated avidin (1:100 dilution; Vector Laboratories, Inc., Burlingame, CA, USA) in a buffer composed of 0.1 M NaHCO_3_ and 0.15 M NaCl, pH 8.2–8.5, for 10 min at room temperature. An avidin/biotin blocking step was performed by incubating slides for 15 min with avidin and then biotin (Avidin/Biotin Blocking Kit, code SP-2001, Vector Laboratories, Inc., Burlingame, CA, USA) at room temperature. The second secondary, biotinylated anti-rabbit Ab (for Von Willebrand Factor; 1:200 code: BA-1000 Vector laboratories, Inc., Burlingame, CA, USA) was applied and incubated for 30 min at room temperature, and slides were then incubated with fluorescein-conjugated avidin (1:100 dilution in 0.1 M NaHCO_3_ and 0.15 M NaCl, pH 8.2–8.5, Vector Laboratories) for 10 min at room temperature. 

### 2.4. Quantification of Mast Cell Density and Microvessel Density

Quantification of mast cell density (MCD) was carried out on TB-stained sections, as well as on tryptase- and c-Kit-immunostained sections, according to a previously described method (“hot-spots”), which is the most widely used method to evaluate MCD in both human and veterinary literature [21,38,39,40]. This method was performed by selecting three intraglandular/intratumoral and three periglandular/peritumoral fields in areas with the highest MC population (“hot spots”), identified by scanning sections at low power (100X magnification). Individual MCs were then counted at 200X magnification, with each microscope field corresponding to an area of 0.785 mm^2^. The same method was used for quantification of microvessel density (MVD), according to literature [39]. Transversally sectioned microvessels with a single layer of endothelial cells were considered, whereas multilayered vessels or those with muscular wall were excluded. As the microvessel diameter is smaller than the distance between adjacent fields, only one transversally sectioned microvessel, as well as only microvessels totally included in the field area, were counted [39].

### 2.5. Statistical Analysis

Data are presented as the mean ± standard deviation (SD) for parametric data, and median for non-parametric data. One way-ANOVA and Kruskal–Wallis test with post-hoc analysis were performed for multiple comparisons, as far as parametric and non-parametric data were concerned, respectively. Unpaired and paired *t* tests were used for single comparisons, with paired *t* test being especially used for comparing MCD between intraglandular/intratumoral and periglandular/peritumoral areas. The differences between areas were considered significant with *p* < 0.05. Pearson or Spearman tests were used for correlations between parametric and non-parametric data, respectively. Analyses were performed using GraphPad Prism 7.

## 3. Result

### 3.1. Histology and Immunohistochemistry

The most common histological subtype of PC was represented by the small acinar subtype (3/8), followed by cribriform (2/8), solid (1/8), signet ring (1/8), and papillary (1/8) subtypes. 

**Figure 1:**
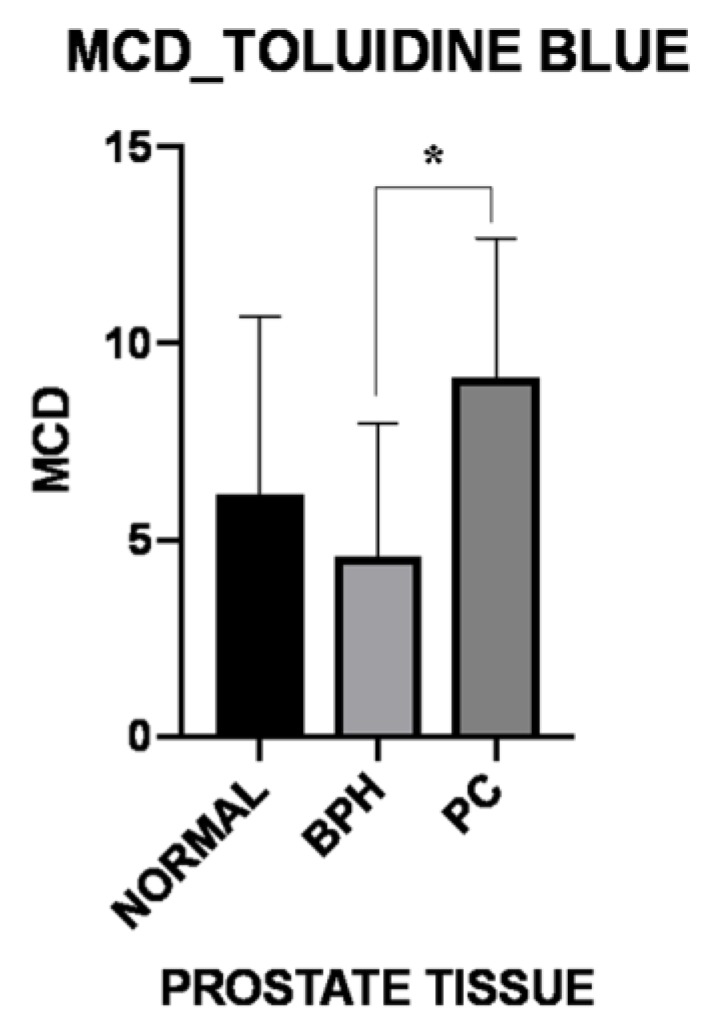
Total (periglandular/peritumoral and intraglandular/intratumoral) mast cell density (MCD) evaluated in Toluidine Blue-stained sections of normal, benign prostate hyperplasia (BPH) and prostate carcinoma (PC) samples. The graph shows an increased MCD in the PC group, compared to the other groups. The asterisk indicates significance (*p* = 0.033) of the comparison between BPH and PC.

When TB-stained sections were considered, an increased, total (peritumoral and intratumoral) MCD was observed in the PC group (8.62 ± 2.67), when compared with total (periglandular and intraglandular) MCD of normal (5.99 ± 4.57; *p* = 0.363) or BPH (4.57 ± 3.37; *p* = 0.033) group, although statistical significance was only reached in the comparison between BPH and PC groups Figure 1.

**Figure 2:**
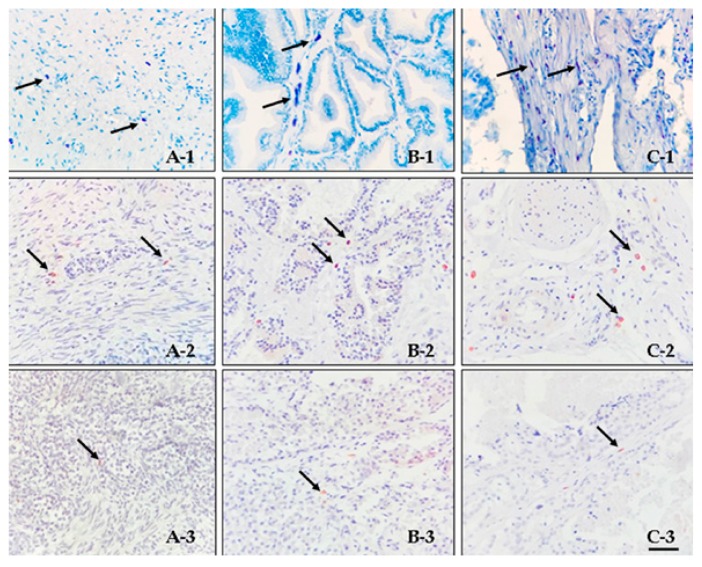
Mast cells (MCs) (indicated by arrows) in canine prostate tissues. (**A**)—normal; (**B**)—benign prostate hyperplasia (BPH); (**C**)—prostate carcinoma (PC). 1-Toluidine Blue (TB) staining: (**A-1**) Scattered MCs as single elements in normal periglandular stroma; (**B-1**) small groups of MCs in BPH intraglandular stroma; (**C-1**) numerous MCs detected in peritumoral stroma. 2-Tryptase immunostaining: scattered, trypase-positive MCs in periglandular and intraglandular stroma of normal (**A-2**) and BPH (**B-2**) samples, respectively, as well as in the peritumoral stroma of a PC case (**C-2**). 3-C-Kit immunostaining: scattered, c-Kit-positive MCs in periglandular and intraglandular stroma of normal (**A-3**) and BPH (**B-3**) samples, respectively, as well as in the peritumoral stroma of a PC case (**C-3**). No c-kit immunostaining is evident in prostate cells in each case. Bar = 50 μm.

MCs were predominantly detected in periglandular/peritumoralareas of TB-stained sections in both normal and PC groups. reaching statistical significance (Table 1). Some differences in MC morphology were also observed depending on their different locations. In particular, MC located in periglandular/peritumoral areas usually showed a more elongated shape, with variably granulated cytoplasm, and they were predominantly detected in close proximity to blood vessels. On the other hand, they were organized in small to medium clusters and exhibited a round to oval shape, with variably granulated cytoplasm, in the intraglandular/intratumoral areas, particularly in the BPH group Figure 2.

As far as tryptase- or c-Kit-immunostained sections were concerned (Figure 2), an increased MCD was observed in periglandular/peritumoral areas, when compared to intraglandular/intratumoral areas in all groups. This finding was particularly evident when tryptase immunoexpression was considered, reaching statistical significance (*p* = 0.034) (Table 2). 

On the other hand, as far as intraglandular/intratumoral areas were concerned, MC number was usually higher in intraglandular areas in BPH samples showing a low (4/15 cases) to moderate (7/15 cases) degree of inflammatory infiltration, when compared to BPH samples without inflammatory infiltration (4/15 cases), although a statistical evaluation was not feasible due to the low number of cases.

Levels of tryptase and c-Kit immunoexpression were significantly associated in periglandularareas of normal tissues (*p* = 0.031), as well as in both periglandular (*p* = 0.033) and intraglandular (*p* = 0.039) areas of BPH. On the other hand, their levels were not significantly associated in peritumoral and intratumoral (*p* = 0.054 and *p* = 0.531, respectively) areas of PC samples. 

In addition, a positive correlation between tryptase and c-Kit immunostaining was observed in periglandular areas of BPH samples (ρ = 0.64 *p* = 0.015). As well, a tendency to a similar positive correlation was observed in peritumoral areas of PC cases, although without reaching statistical significance (ρ = 0.59 *p* = 0.432). On the other hand, a strong correlation for c-Kit immunoexpression was observed between intraglandular/intratumoral and periglandular/peritumoral areas in both BPH and PC cases (ρ = 0.75 *p* = 0.031 and ρ = 0.57 *p* = 0.024, respectively). 

MCD levels based on tryptase and c-Kit immunohistochemical expression in intraglanular/intratumoral versus periglandular/peritumoral areas are summarized in Table 2 and Table 3, respectively. 

### 3.2. Double Immunofluorescence

MCs were mainly organized in small clusters (2–3 cells), which were predominantly detectable in close proximity to blood vessels in all groups examined. (Figure S1 see Supplementary File).

### 3.3. Microvessel Density

**Figure 3:**
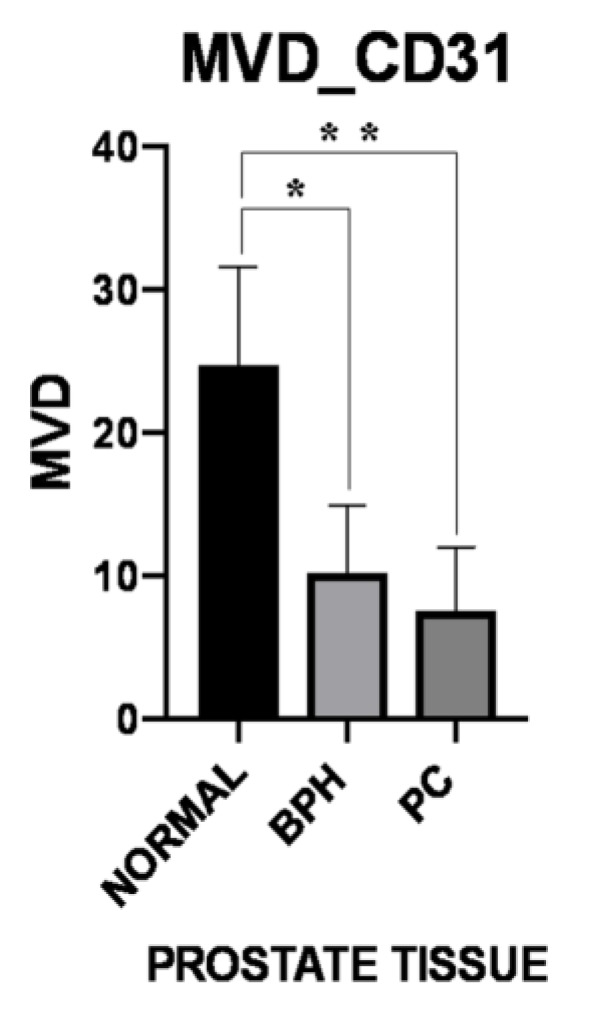
Microvessel density (MVD) based on CD31 immunostaining in prostate samples. The graph shows a significantly higher MVD in normal group, when compared with benign prostate hyperplasia (BPH) (single asterisk: *p* = 0.015) and prostate carcinoma (PC) (two asterisks: *p* = 0.003) groups.

**Figure 4:**
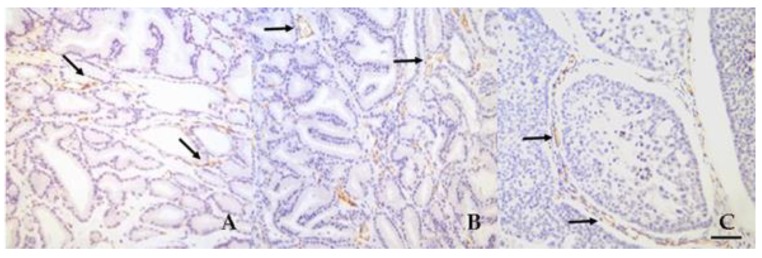
CD31-stained microvessels (indicated by arrows) in normal prostate tissue (**A**), benign prostate hyperplasia (**B**), and prostate carcinoma (**C**). Multiple, brown-stained microvessels are visible in intraglandular (**A**,**B**) and intratumoral (**C**) areas. Bar = 40 μm.

MVD evaluated on the basis of CD31 immunostaining showed a higher MVD in normal samples (24.7 ± 6.92), when compared with BPH and PC (10.19 ± 4.72 and 7.60 ± 4.41, respectively) groups, reaching statistical significance (normal versus BPH: *p* = 0.015; normal versus PC: *p* = 0.003). (Figure 3 and Figure 4). Microvessels appeared to be uniformly distributed throughout tissues, without differences in MVD between periglandular/peritumoral and intraglandular/intratumoral areas in BPH and PC groups. On the other hand, MVD was significantly higher (*p* = 0.047) in periglandular areas when compared to intraglandular areas in normal samples Table 4.

**Figure 5:**
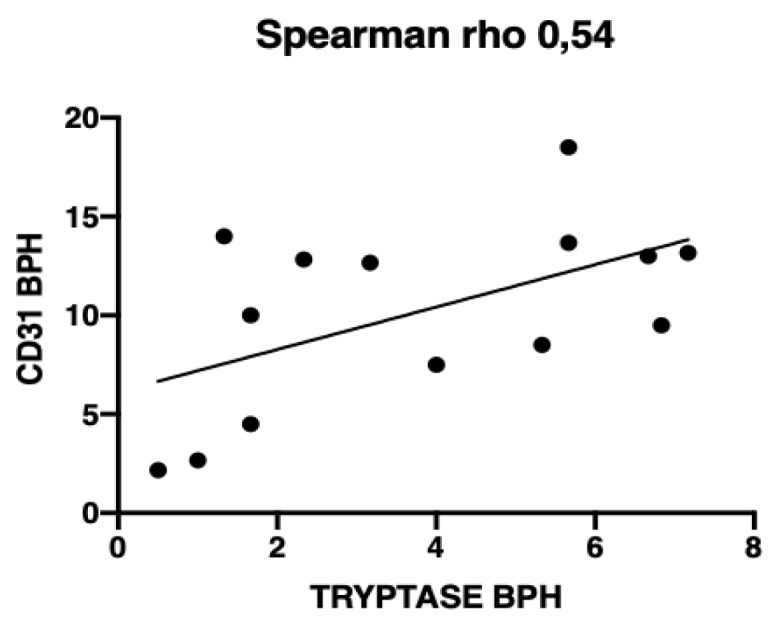
The Spearman’s Rho diagram showing correlation between CD31 immunostaining-based microvessel density (MVD) and tryptase immunostaining-based mast cells density (MCD) in benign prostate hyperplasia (BPH) samples (ρ = 0.54 *p* = 0.041).

A significant positive correlation between MVD and tryptase immunostaining-based MCD was observed in BPH samples (ρ = 0.54 *p* = 0.041), whereas these parameters were not significantly correlated in normal and PC cases (ρ = 0.60 *p* = 0.314 and ρ = −0.45 *p* = 0.262, respectively) Figure 5.

## 4. Discussion

This study aimed to describe the distribution, tryptase and c-Kit immunoexpression, and the possible correlation with MVD of MC populations in canine prostate tissues. Many studies are available in the human literature investigating MC populations in various organs and tumors [17], with several researches concerning human prostate tissue [22,23,24,25,41,42,43]. In contrast, as far as the veterinary literature is concerned, only a few studies have been carried out in order to evaluate MC distribution and morphological features in normal organ and tissues [44,45,46,47,48], or to investigate MCD in neoplastic conditions [35,39,49,50,51,52], especially in relation to angiogenesis [35,47,51], and no information is available regarding prostate tissue.

As far as the localization pattern of MCs within tissues is concerned, MCs were found to be located near blood and lymphatic vessels in dogs [48]. In previous human studies, MCs were also mainly found adjacent to blood and lymphatic vessels in various tissues [17,48], including the prostatic stroma [53,54]. Likewise, MCs were mainly detected in the stromal compartment of prostate tissue in our cases, with a predominant distribution in close proximity to blood vessels in all groups examined. This localization probably reflects the role of MCs as the first cells to initiate reactive responses, even towards neoplastic cells [17].

TB-stained sections allowed to evaluate the presence and distribution of the total MC population in the samples examined. TB-based MCD was significantly increased in PC cases when compared to BPH and normal groups. This result parallels with several previous studies and confirms that solid cancers, including PC, are commonly infiltrated by a high number of innate and adaptive immune cells [42]. In particular, among innate immune cells, MC infiltration has been frequently observed in human PC [23,24,25,31,42]. In this respect, MCs can positively or negatively regulate tumor growth, depending on the tumor type and microenvironment [21,31,55]. In fact, MC degranulation can recruit immune cells, thus stimulating an anti-tumor immune response [18,23,31]. On the other hand, MCs can exert a pro-tumor effect by promoting angiogenesis and modulating the extracellular matrix [31,56,57]. As a consequence, there are conflicting reports concerning the role played by MCs in prostate tissue [22,23,25], as well as on their possible positive or negative prognostic significance [24,42]. The different results obtained in the various studies carried out on human PC may be due to the differences in the study method, including MC markers evaluated (tryptase versus c-Kit versus chymase), the area considered (intratumoral versus peritumoral), and the type of tissue sample (biopsy versus tissue microarrays) [23]. Therefore, a better definition of the study method aimed to identify the extent and distribution of MC distribution in PC tissues is necessary in order to clearly define the role played by MCs in prostate cancer. 

Analyzing in detail the distribution of MC population by means of tryptase immunostaining, a predominant localization in periglandular/peritumoral areas in normal, BPH and PC cases was found, in comparison to intraglandular/intratumoral areas. Thus, the present results clearly demonstrate similar MC distribution patterns between human [24,41,43] and canine prostate tissues, which could reflect similar roles played by MCs in both human and canine prostate.

A slight increase in MC number was also observed in intraglandular/intratumoral areas in BPH, when compared to the other two groups, which could be due to the presence of inflammatory infiltration observed in some BPH samples [21,43,58,59]. A similar increase was found for c-Kit immunostaining, as previously reported by Globa et al. [21].

C-Kit is a tyrosine kinase protein type III, and its ligand SCF play a key role in MC biology [17,36,60,61]. In fact, SCF represents an essential factor for MC survival and development and mice lacking c-Kit or its ligand result in absence of MCs [17,36,60,61]. In our study, the frequent c-Kit immunoexpression detected in prostate MC populations, particularly in BPH and PC groups, give support to the importance of c-Kit in regulating MC survival and activation [17,36,60,61]. On the other hand, a few works have investigated the role of c-Kit expression in human [62] and canine [36] PC cells itself, reporting a small number of c-Kit-positive PC samples [36,62]. In this respect, c-Kit-positive prostate epithelial cells were not detected in the normal samples considered in the present study, and only a very low positivity in scattered prostate cells was observed in the other groups, thus confirming the hypothesis that c-Kit expression in PC cells may be restricted to a limited number of cases, not included in the PC samples selected for the present work. 

Tryptase is one of the serine proteases secreted by MCs. A correlation between MC tryptase expression and increased neovascularization has been detected in different human solid tumors, including PC, indicating a role for this protease in angiogenesis after its release from activated MCs [63]. Conversely, in the present study, a positive correlation between tryptase immunostaining-based MCD and MVD was observed in BPH but not in PC cases, thus suggesting that tryptase does not exert an angiogenic activity in canine PC. 

As far as MVD based on CD31 immunostaining is concerned, it showed high levels in the normal group of canine prostates included in our study. This finding could be related to the young age of the dogs, thus allowing to suppose that canine prostate tissue may need a rich vascularization to complete its development. On the other hand, the different correlations between MCD and MVD observed in the present study gives further support to the hypothesis that MCs may influence angiogenesis in canine BPH, but not in tumor tissue. In fact, although correlations between MCD and MVD have been reported in canine mammary carcinoma [35], transmissible venereal tumor [51], melanoma [49], hemangioma, hemangiosarcoma [52] and nodal lymphoma [39], MVD was not increased in canine PC when compared with normal tissue or BPH, similarly to humans [64,65,66]. This finding could reflect the similarities between canine PC and human advanced, hormone-refractory prostate cancer, since PC is usually diagnosed at an advanced stage and is not responsive to castration in dogs [27,29]. In this respect, it is important to highlight that MCs are supposed to exert pro-tumorigenic effects in the early stages of human PC, whereas they may represent anti- or not-tumorigenic factors in the late PC stage, as a consequence of increased inflammatory anti-tumor reaction, induction of apoptosis, inhibition of cell growth or prevention of neovascularization [12,67,68]. However, opposing results reporting an increased MVD in prostate cancer also exist in human literature, which may be due to the different methods and markers used to evaluate MVD. In fact, since CD31 is considered to be more specific than other endothelial cells markers, such as CD34, von Willebrand factor or CD105, MVD usually results to be lower when determined by using anti-CD31 Abs in comparison to the Abs directed against the other markers [64,69]. These controversial results thus suggest the need for a standard method to be used to evaluate MVD in tumors, allowing a better comparison between different studies carried out in both human and canine species. 

## 5. Conclusions

This is the first study investigating MC population in canine prostate tissues. Although preliminary, our results suggest that MCs may play an important role in tumor-microenvironment interaction in canine PC. However, further studies carried out on a higher number of cases are necessary to perform a more detailed MC analysis, including investigations of the expression of other MC proteases, such as chymase, as well as a comparison between MCD and clinical follow up of the canine PC patients, in order to find a possible relationship with canine PC prognosis. In addition, a consensus on the study methods used to evaluate MCD and MVD would be necessary for future researches, in order to allow better comparisons between different studies carried out in both human and canine species.

## Figures and Tables

**Table 1:** Mast cell density (MCD) in periglandular/peritumoral areas versus intraglandular/intratumoral areas based on Toluidine Blue staining. *p*-Value is significant in normal and prostate carcinoma (PC) groups, but not in benign prostate hyperplasia (BPH) group.

MCD-Toluidine Blue	Normal	BPH	PC
Periglandular/peritumoral	9.39 ± 7.38	4.89 ± 3.46	15.50 ± 5.81
Intraglandular/intratumoral *p*-Value	2.61 ± 2.47 0.031	4.27 ± 3.91 0.365	2.75 ± 2.89 0.007

**Table 2:** Mast cell density (MCD) in periglandular/peritumoral areas versus intraglandular/intratumoral areas based on tryptase immunoexpression. *p*-Value is significant in all the three groups: normal, benign prostate hyperplasia (BPH) and prostate carcinoma (PC).

MCD-Tryptase	Normal	BPH	PC
Periglandular/peritumoral	6.11 ± 1.55	4.84 ± 0.80	9.37 ± 1.89
Intraglandular/intratumoral *p*-Value	2.22 ± 0.98 0.003	2.82 ± 0.60 0.034	1.91 ± 0.80 0.031

**Table 3:** Mast cell density (MCD) in periglandular/peritumoral areas versus intraglandular/intratumoral areas based on c-Kit immunoexpression. *p*-Value is significant in benign prostate hyperplasia (BPH) and prostate carcinoma (PC) groups.

MCD-c-Kit	Normal	BPH	PC
Periglandular/peritumoral	1.05 ± 0.49	3.20 ± 0.88	6.10 ± 1.40
Intraglandular/intratumoral *p*-Value	0.72 ± 0.30 0.875	1.29 ± 0.37 0.023	1.00 ± 0.35 0.004

**Table 4:** Microvessel density (MVD) in periglandular/peritumoral versus intraglandular/intratumoral areas based on CD31 immunostaining.

MVD-CD31	Normal	BPH	PC
Periglandular/peritumoral	18.06 ± 4.10	10.69 ± 4.70	8.00 ± 4.16
Intraglandular/intratumoral *p*-Value	31.30 ± 11.80 0.047	9.68 ± 5.24 0.193	7.16 ± 4.96 0.398

## Supplementary Materials

The following are available online at http://www.mdpi.com/2306-7381/6/1/16/s1, Figure S1: double immunofluorescence.
