# Supplementary material for: Tumor–Microenvironment Interaction: Analysis of Mast Cell Populations in Normal Tissue and Proliferative Disorders of the Canine Prostate
Source: Vet Sci. 2019 Feb 13;6(1):16. doi: 10.3390/vetsci6010016 (PMC6466327; doi:10.3390/vetsci6010016)
Supplement: Supplementary file 1 [file vetsci-06-00016-s001.pdf]

veterinary sciences

Supplementary file: Tumor–Microenvironment Interaction: Analysis of Mast Cell Populations in Normal Tissue and Proliferative Disorders of the Canine Prostate

Sabrina Vanessa Patrizia Defourny [1], Mariarita Romanucci [1], Valeria Grieco [2], Gina Rosaria Quaglione [3], Chiara Santolini [4] and Leonardo Della Salda [1,*]

1 Faculty of Veterinary Medicine, University of Teramo, 64100 Teramo, Italy; sdefourny@unite.it (S.V.P.D.); mromanucci@unite.it (M.R.)
2 Department of Veterinary Medicine, University of Milan, 20154 Milan, Italy; valeria.grieco@unimi.it
3 Unità Ospedaliera Complessa, Anatomia patologica, Ospedale G. Mazzini, 64100 Teramo, Italy; gina.quaglione@aslteramo.it
4 Veterinary practitioner, 63812 Montegranaro, Italy; chiasan@alice.it
* Correspondence: ldellasalda@unite.it; Tel.: +39-861-266866

Received: 4 December 2018; Accepted: 7 February 2019; Published: 13 February 2019

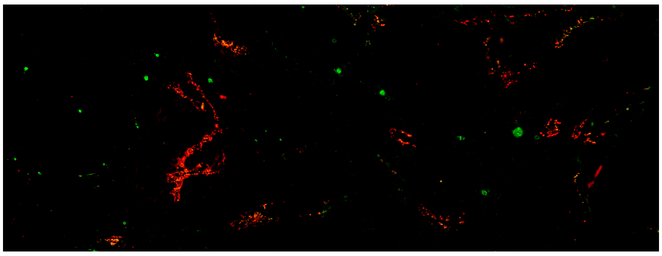

A B

Figure S1 Double immunofluorescence. Tryptase-Von Willebrand Factor showing small cluster of mast cells (green) detectable in close proximity to blood vessels (red) in periglandular (**A**) and intraglandular (**B**) areas of benign prostate hyperplasia
